# Pseudomonas pharyngitis in a cystic fibrosis patient

**DOI:** 10.1002/rcr2.325

**Published:** 2018-04-27

**Authors:** Shyamala Pradeepan, Peter Wark

**Affiliations:** ^1^ Department of Respiratory and Sleep Medicine John Hunter Hospital New Lambton Heights NSW Australia; ^2^ University of Newcastle Callaghan NSW Australia; ^3^ Hunter Medical Research Institute Newcastle NSW Australia

**Keywords:** Cystic fibrosis, Pseudomonas pharyngitis, sinusitis

## Abstract

We report a case of Pseudomonas pharyngitis in a cystic fibrosis (CF) patient not known to have prior colonization of Pseudomonas. We believe that this is one of the first cases of pseudomonas pharyngitis in a CF patient.

## Abstract 2

We report a case of Pseudomonas pharyngitis in a cystic fibrosis (CF) patient not known to have prior colonisation of Pseudomonas. We believe that this is one of the first cases of pseudomonas pharyngitis in a CF patient.

## Introduction

This is a case study of a patient with cystic fibrosis (CF) who presented with features of recurrent sore throat to a routine outpatient CF clinic.

## Case Report

A 23‐year‐old male with CF, genotype F508del and c.350G > A, presented to a scheduled CF clinic visit. He is a student from a local University.

He had minimal respiratory symptoms. He had methicillin‐sensitive staphylococcal aureus isolated on a prior occasion with no pseudomonas identified. Chest X‐ray showed early signs of bronchiectasis. He had no features of chronic sinusitis. He had impaired glucose tolerance with normal glycaemia and had no evidence of pancreatic insufficiency or any liver disease.

On presentation, he complained of four episodes of sore throat, each lasting up to 4–5 days, resolving spontaneously in the prior 3 months. He had no symptoms of nasal congestion, sinus pain, or postnasal drip and no cough or production of sputum. He had not had any treatment for it. At the time of review, he was systemically well with no fevers. His swallow was normal. He had not had any changes to his living environment or any close contact with other CF patients.

On examination, he was afebrile, and PR was 86 and BP 110/80 mmHg. His BMI was 22.

The throat examination revealed an inflamed pharynx with no purulent discharge, and his tonsils were not enlarged or inflamed. Cervical and submandibular nodes were not tender or enlarged. His lungs were clear. His FEV1 at that visit was 4.28 L/min (87% predicted).

A throat swab was obtained as part of assessment for standard bacterial culture, and a nasopharyngeal swab was also taken for respiratory viral PCR. He did not have any sputum to produce for testing at this visit. He did have a sputum sample taken 6 weeks later, which was negative for Pseudomonas.

The bacterial culture of the throat swab isolated non‐mucoid Pseudomonas aeruginosa, which was sensitive to Ciprofloxacin, Gentamycin and Piperacillin, and Tazobactam. The respiratory viral PCR was negative. Based on these results, he was started on Pseudomonas eradication therapy, with oral ciprofloxacin and nebulized tobramycin. His symptoms of sore throat disappeared with treatment, but his repeat throat swab after intended eradication still grew Pseudomonas. At this point, he was referred to an ENT surgeon after CT sinuses, which returned features of mild chronic sinusitis.

He underwent tonsillectomy, septal reconstruction, turbinectomy, and full FESS. At surgery, a sample taken from the right maxillary sinus also grew Pseudomonas. He was then treated with intravenous Piperacillin, Tazobactam, and oral Ciprofloxacin for 4 weeks.

Almost 6 months after the initial isolation, his throat swabs returned negative for Pseudomonas. He did not have any more clinical features of pharyngitis.

He made good recovery clinically from pharyngitis and sinusitis and continues to follow up with our CF service. He remains free of Pseudomonas on sputum cultures to date but has isolated Staphylococcus in current sputum.

## Discussion

CF is a multisystem disease affecting the lungs, digestive system, sweat glands, and the reproductive tract. Patients with CF have abnormal transport of chloride and sodium across secretory epithelia, resulting in thickened, viscous secretions in the bronchi, biliary tract, pancreas, intestines, and reproductive system. Although the disease is a multisystem disorder, progressive lung disease continues to be the major cause of morbidity and mortality for most 1.

Although CF is a multisystem disorder, isolated pharyngitis is not a recognized problem or reported commonly. Features of pharyngitis are seen associated with inter‐current viral infections. Isolated pharyngitis is not reported in CF literature. Sore throat or pharyngitis has not been typically described in chronic sinusitis or postnasal drip. On the other hand, the majority of CF patients develop sinus disease. Radiographs reveal pan opacification of the paranasal sinuses in 90–100% of patients older than 8 months of age, and nasal polyposis is seen in 10–32% of patients 2. Sinus infections can trigger lower respiratory exacerbation in some patients, although organisms found in the sinuses do not always match those recovered from the lungs.

There are several studies looking at routine throat swabs for the isolation of pathogens in CF mainly in the paediatric populations. A recent study looked at throat swabs as a marker of lower‐airway Pseudomonas colonization 3. This study showed that both sputum and throat swab were 100% sensitive in predicting BAL Pseudomonas, whereas the negative predictive value of a throat swab was only 50%. Zemanick et al., in a 2015 study, suggest poor correlation between throat swab isolation and the Pseudomonas from induced sputum. A prior case report suggested the presence of a positive throat swab in a patient colonized with Pseudomonas of the sinuses 4. This patient had existing sinus disease and no clinical features of recurrent pharyngitis.

This case illustrates the importance of obtaining throat swabs in clinically symptomatic patients for diagnosis and offering appropriate treatment of the offending pathogen for pharyngitis. This also illustrates the possibility of finding the offending pathogens of sinuses via throat swabs in adult CF patients. This also highlights the importance of early detection of Pseudomonas for early eradication as Pseudomonas is linked to recurrent exacerbations and deteriorating lung function with worsening bronchiectasis 5. Throat swabs are taken from paediatric patients with CF in a regular fashion but not routinely in adult patients. There is potential for this to be performed regularly to investigate for the presence of pathogens in the sinus cavity and as a marker of eradication of sinus infection/colonization. The sinus infection is a nidus for airway infection, and hence, the need for early detection and eradication becomes an important aspect of treatment. Nasal clearance surgery should also be considered for the eradication of pathogens in the sinuses.

## Disclosure Statement

Appropriate written informed consent was obtained for publication of this case report.
